# Effect of Dual Bronchodilation on the Exercise Capacity of Individuals With Non-Cystic Fibrosis Bronchiectasis: Protocol for a Randomized Controlled Double-Blind Crossover Study

**DOI:** 10.2196/68582

**Published:** 2025-07-28

**Authors:** Cibele Cristine Berto Marques da Silva, Simone Dal Corso, Adriana Claudia Lunardi, Alfredo José Fonseca, Samia Zahi Rached, Rodrigo Abensur Athanazio, Celso Ricardo Fernandes Carvalho

**Affiliations:** 1 Department of Physical Therapy University of São Paulo São Paulo Brazil; 2 Department of Allergy, Immunology and Respiratory Medicine Monash University Monash Australia; 3 Medical Clinic Department, Clinics Hospital University of São Paulo São Paulo Brazil; 4 Pneumology Department, Heart Institute University of São Paulo São Paulo Brazil

**Keywords:** bronchiectasis, bronchodilator agents, exercise test, exercise, non-cystic fibrosis bronchiectasis, exercise capacity, thoracoabdominal kinematics

## Abstract

**Background:**

Bronchodilators (BDs) have been used therapeutically to improve exercise capacity in patients with other chronic respiratory diseases. However, the effect of BDs on the exercise capacity of individuals with non-cystic fibrosis bronchiectasis (NCFB) is poorly understood.

**Objective:**

The aim of this study was to evaluate the effects of BDs on exercise capacity and thoracoabdominal kinematics in patients with NCFB.

**Methods:**

This crossover randomized controlled trial will involve 45 outpatients with NCFB aged 18 to 59 years. They will be evaluated in 3 visits. On day 1, the maximal exercise capacity (cardiopulmonary exercise test; peak work rate [W_peak_]) will be assessed. On day 2, individuals will be randomized to receive either BD (ipratropium bromide 160 µg and fenoterol hydrobromide 400 µg) or a placebo and then undergo simultaneous endurance exercise capacity (constant work-rate exercise test) and thoracoabdominal kinematics (optoelectronic plethysmography) assessments. After at least 1-week washout (day 3), the individuals will repeat the same assessments as on day 2 in the reverse order. The time to the limit of tolerance will be obtained in both groups (BD and placebo groups) as the primary outcome. Thoracoabdominal kinematics will be assessed at 3 time points: at rest, during unloaded exercise, and at 75% W_peak_. The total chest wall and compartmental volumes as well as thoracoabdominal asynchrony will be assessed. The assessors and patients will be blinded to the interventions (BDs or placebo). Data will be compared using 1-sided *t* tests or Wilcoxon tests and repeated-measures analysis of variance or Friedman tests. Categorical data will be analyzed using the chi-square test or Fisher test. The associations among variables will be analyzed using Pearson or Spearman correlation. The significance level will be set at 5% (*P*<.05).

**Results:**

The ethics approval was granted in November 2018, and a pilot study was commenced in April 2019 but was interrupted due to the COVID-19 pandemic. The study restarted in April 2022, and data collection is anticipated to continue until November 2025. The publication of the results is anticipated to be in 2025 or 2026.

**Conclusions:**

There is no evidence that BDs can improve the exercise capacity of patients with NCFB. This trial will compare the endurance exercise capacity of the same individual with and without dual bronchodilation. If successful, this study will demonstrate that exercise capacity can be improved with the use of BDs in adults with NCFB.

**Trial Registration:**

ClinicalTrials.gov NCT05183841; https://clinicaltrials.gov/study/NCT05183841

**International Registered Report Identifier (IRRID):**

DERR1-10.2196/68582

## Introduction

Non-cystic fibrosis bronchiectasis (NCFB) is radiologically characterized by permanent dilation of the bronchi and clinically characterized by productive cough and recurrent respiratory infections [1]. NCFB is becoming increasingly prevalent and can affect individuals of any age [2]. The pathophysiological mechanisms include persistent bacterial infections, dysregulated immune responses, impaired mucociliary clearance, and airway obstruction—all of which can cause long-term lung impairment (vicious vortex) [1].

Although the optimal therapy for NCFB remains unknown, various pharmacological strategies have been proven beneficial for improving both the quality of life and clinical outcomes. Anti-inflammatory treatment seems to reduce the volume of secretions and inflammatory markers in the sputum and improve the quality of life [3]. In general, antibiotics effectively reduce the bacterial load in the airways [4]. Although the role of bronchodilators (BDs) in the treatment of NCFB is less clear, BDs appear to improve lung function in some patients [5]. Neither antibiotics nor anti-inflammatories can significantly improve the lung function of individuals with NCFB [6], making BDs a therapeutic alternative.

The use of BDs for other chronic respiratory diseases such as asthma and chronic obstructive pulmonary disease (COPD) is already well established and recommended in their respective guidelines [7,8]. According to the Global Initiative for Chronic Obstructive Lung Disease [8], the use of BDs (long-acting β-2 agonists or muscarinic antagonists) is indicated for individuals with severe disease, symptoms, or exercise limitations, as they reduce airway resistance, promote lung deflation, and enable better alveolar ventilation, both at rest and during exercise, in this population.

Despite the lack of robust studies demonstrating the effectiveness of BD use in the treatment of NCFB, BDs continue to be among the most commonly prescribed medications worldwide for this population [9,10]. A small previous study in adults with clinically stable NCFB revealed that combining inhaled corticosteroids with long-acting β-2 agonists might improve dyspnea and increase the number of cough-free days compared with high-dose inhaled corticosteroids, with no effect on the quality of life, number of hospitalizations, or lung function [11]. Despite being a randomized trial, neither patients nor assessors were blinded, and there was an overall high risk of bias, according to Goyal and Chang [12]. A more recent and larger study [13] evaluated the effects of 6-month tiotropium (long-acting muscarinic antagonists) treatment in 90 clinically stable adults with NCFB. Compared with placebo, tiotropium improved the forced expiratory volume in the first second (FEV1) by 58 mL and the forced vital capacity (FVC) by 78 mL. Nevertheless, there was no difference in the quality of life, chronic cough, exercise capacity, or neutrophil and eosinophil counts in the blood or sputum. Furthermore, it did not reduce the number of exacerbations. To date, there are no studies on the effect of BDs on the exercise capacity of this population.

In individuals with NCFB, airflow limitation and dyspnea are strongly associated with exercise capacity [14]. It seems plausible that BDs may improve the exercise capacity of individuals with NCFB similar to what has been observed in individuals with COPD. Additionally, thoracoabdominal asynchrony (TAA) is very prevalent in individuals with COPD and is associated with exercise limitations [15,16]. In this population, the main effect of BDs is an increase in the basal inspiratory capacity and changes in dynamic hyperinflation during exercise, which improves exercise capacity [17]. However, no study has evaluated TAA or the thoracoabdominal kinematics of individuals with NCFB. Therefore, we hypothesize that dual bronchodilation can improve the exercise capacity of individuals with NCFB and reduce TAA during exercise.

## Methods

### Study Design

**Figure 1:**
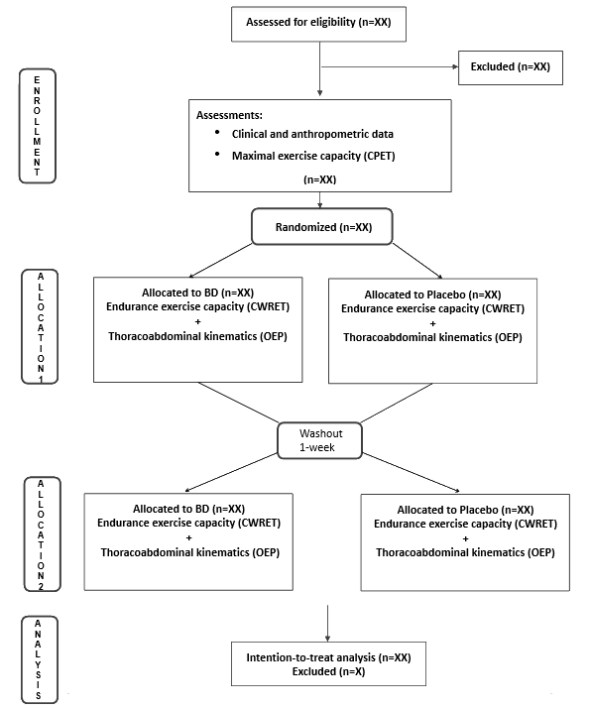
CONSORT (Consolidated Standards of Reporting Trials) diagram showing the flow of participants through each stage of the randomized crossover trial. BD: bronchodilator; CPET: cardiopulmonary exercise test; CWRET: constant work-rate exercise test; OEP: optoelectronic plethysmography.

This is a randomized, placebo-controlled, double-blind, crossover trial (Figure 1). Participants are assigned in a 1:1 ratio to 2 treatment periods, each lasting 1 hour, with a 1-week washout period between them.

### Ethical Considerations

Patients will be recruited from an outpatient tertiary clinic specializing in treating patients with NCFB at Hospital das Clínicas, Universidade de São Paulo, São Paulo-Brazil. The Hospital Research Ethics Committee of the University of São Paulo approved the study (approval 3.008.886). All participants will provide written informed consent prior to data collection. The data will be deidentified by excluding the main identifying elements such as name, address, date of birth, phone number, email, medical record number, institutional identification number, and social security number. Participants will receive an identification code, according to the order of inclusion in the study. The participants will be informed about the possibility of withdrawing at any time, without loss of their treatment at the institution. Additionally, the participant will be informed about the voluntary nature of their participation. Financial assistance for transportation will be available. This study is registered on ClinicalTrials.gov as NCT05183841.

### Eligibility Criteria

Individuals aged 18-59 years, of either sex, diagnosed with NCFB on the basis of features on the computed tomography scan of the thorax [18], clinically stable (last 30 days without exacerbation/hospitalization), with trapped air on body plethysmography, that is, if residual volume/total lung capacity ratio is higher than 120% of the predicted [19,20], and physically inactive (<60 minutes of structured or planned physical activity per week) [21] will be eligible to participate. The exclusion criteria will be as follows: smokers or former smokers (>10 pack-years); other concomitant respiratory diseases, and cardiovascular or musculoskeletal diseases that may interfere with the patient’s evaluations; uncontrolled arterial hypertension or diabetes; and pregnant or breastfeeding. Patients who have participated in a pulmonary rehabilitation program in the last 6 months or are participating in another research protocol as well as those with cognitive deficits that limit comprehension will also be excluded.

### Experimental Design

**Figure 2:**
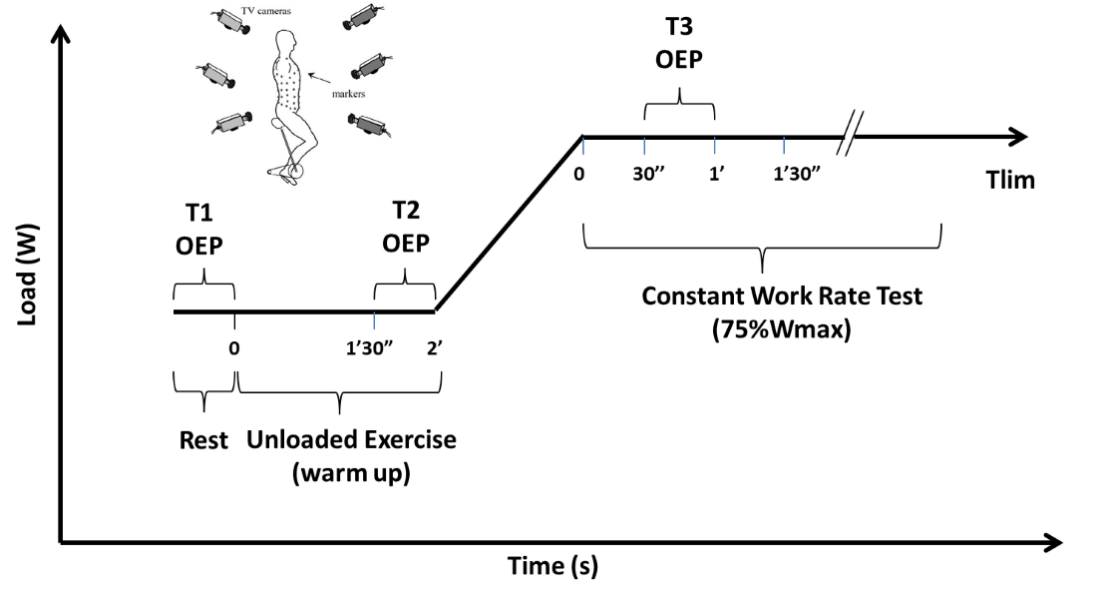
Experimental design. Assessment of endurance exercise capacity simultaneously with thoracoabdominal kinematics. OEP: optoelectronic plethysmography; T1: thoracoabdominal kinematics assessment at rest; T2: thoracoabdominal kinematics assessment at unloaded exercise; T3: thoracoabdominal kinematics assessment at 75% Wpeak; Tlim, time to the limit of tolerance; TV: television; Wmax: maximal or peak work rate.

Patients will be evaluated on 3 nonconsecutive days. Clinical data (anthropometrical and bronchiectasis severity assessed by FACED [FEV1% predicted score, Age, Chronic colonization by *Pseudomonas aeruginosa*, Radiological extension, Dyspnea]) [10] will be obtained from the participants’ medical records. On day 1, pulmonary function will be assessed via whole-body plethysmography, and the maximal exercise capacity will be measured via a cardiopulmonary exercise test (CPET) to obtain the peak work rate (W_peak_). On day 2, patients will be randomly allocated to receive BDs (160 µg of ipratropium bromide and 400 µg of fenoterol hydrobromide) or a placebo (norflurane or HFA134A, a harmless propellant), followed by simultaneous assessments of endurance exercise capacity (constant work-rate exercise test, CWRET) and thoracoabdominal kinematics (optoelectronic plethysmography). On day 3, after at least 1-week washout with no maximum deadline, the patient will repeat the same assessment as on day 2 in the reverse order. This washout period was defined according to the maximum BD withholding time (48 hours) [22], aiming to minimize the carryover effect. In case of NCFB exacerbation during the washout period, a 30-day recovery interval will be performed for the next assessment. Spirometric evaluations will be performed pre-CWRET before and after BDs or placebo. The time to the limit of tolerance (T_lim_) will be obtained under both conditions (BDs and placebo). Thoracoabdominal kinematics will be assessed at 3 time points: at rest (T1), during the unloaded exercise (T2), and at 75% W_peak_ obtained from CPET (T3). Thoracoabdominal kinematics will assess volume variables, including total chest wall volume, compartmental volume, and TAA. The assessor and patient will be blinded to the intervention (BDs or placebo). All exercise tests will be performed on a cycle ergometer (Figure 2).

### Pulmonary Function

Spirometry and lung volume measurements will be obtained via a calibrated spirometer (nSpire KoKoUSB Spirometer) and a whole-body plethysmograph (Elite Dx, Elite Series; Medical Graphics Corporation), respectively, according to standardized recommendations [23]. The measurement of the diffusing capacity of the lungs for carbon monoxide will be obtained by the single-breath maneuver in absolute and relative values [24]. The FVC, FEV1, total lung capacity, residual volume, and diffusing capacity of the lungs for carbon monoxide will be expressed as percentages of the predicted values [25-27]. Spirometric evaluations will be performed before CWRET and before and after BDs or placebo, and a change of >10% relative to the predicted value for FEV1 or FVC will be considered a positive response to the BD [24].

### CPET Examination

CPET will be performed using an electrical cycle ergometer (Corival, Lode BV; Medical Technology) linked to a digital system equipped with an exercise evaluation system (CardioO2 System; Medical Graphics Corporation) in accordance with the American Thoracic Society/American College of Chest Physicians guidelines [28]. Peripheral oxygen saturation (SpO_2_), as measured via pulse oximetry (Onyx, Model 9500; Nonin) and electrocardiography (Welch Allyn CardioPerfect, Inc), will be monitored continuously during the tests. The following variables will be recorded breath-by-breath during rest, exercise, and subsequent testing: work rate, oxygen consumption, minute ventilation, carbon dioxide production, respiratory exchange rate, and heart rate. Additionally, blood pressure, SpO_2_, and the modified Borg scores (0-10) for leg discomfort and dyspnea [29] will be measured at rest and every 2 minutes during testing until the end of testing [30]. The participants will perform a ramp symptom-limited CPET consisting of 2 minutes of rest, 2 minutes of warm-up (unloaded pedaling), and an incremental work period (an increase from 10 W/min to 20 W/min, considering the patient’s level of daily activity) [31]. The predicted CPET values will be obtained from the Brazilian population [32]. Although there is no specific information about the threshold at which arterial desaturation becomes hazardous, the test should be terminated if SpO_2_ falls below 80% [33].

### CWRET Analysis

CWRET will be performed using an electrical cycle ergometer after a CPET is completed for an appropriate work rate for CWRET to be estimated [34]. CWRET is selected to be 75%-80% of CPET W_peak_. Continuous monitoring of SpO_2_, heart rate, and modified Borg scores (0-10) for leg discomfort and dyspnea will be performed according to the American Thoracic Society/American College of Chest Physicians guidelines [28]. The target duration for CWRET will be 180 seconds to 480 seconds. For patients not achieving a baseline T_lim_ within 180-480 seconds, the work rate will be adjusted to bring T_lim_ within the desired range (ie, SD 5 W), and the test will be repeated [35].

### Thoracoabdominal Kinematics

Thoracoabdominal kinematics will be evaluated by optoelectronic plethysmography (BTS), as previously described [36]. Briefly, a video is recorded with 8 solid-state charge-coupled cameras operating at 100 frames per second and synchronized with an infrared flashing light-emitting diode. Four cameras will be positioned in front of the participant and 4 behind; 89 retroreflective markers will be placed on the anterior and posterior sides of the trunk according to a previously described protocol [37]. A 3D calibration of the equipment will be performed on the basis of the manufacturer’s recommendations. The assessment will be performed with the individual on a cycle ergometer at 3 time points, that is, at rest, during the unloaded exercise, and at 75% W_peak_, according to the values obtained from CPET. The data analysis will use an average of 6 homogeneous respiratory cycles.

### Allocation, Randomization, and Blinding

Eligible patients will be randomly allocated to either BD or placebo interventions. The randomization sequence will be computer-generated and placed in sealed, opaque, and numbered envelopes by a volunteer not involved in the study. The researcher who will provide the intervention (BD or placebo) will open the envelopes and will not be involved in the data collection or analysis. The participants will not be informed if they will receive BD or placebo intervention. The BD and placebo inhaler devices will be identical to the pressurized metered dose type. All the assessments will be performed by a blinded assessor for the interventions.

### Interventions

Participants will be instructed not to consume alcohol or coffee or perform intense physical activity the day before the exercise test. In addition, they will be oriented to suspend routine BD medication for 6 hours for short-acting β-2 agonists and for 12 hours for short-acting muscarinic antagonists, and for 24 hours for long-acting β-2 agonists and 48 hours for long-acting muscarinic antagonists before the exercise test, according to the European Respiratory Society 2019 guidelines [22]. In case of exacerbation of symptoms during the period of BD suspension, the patient will be advised to use the medication, and the test will be rescheduled.

According to the randomization, a trained respiratory physiotherapist will administer either BD or placebo, ensuring proper treatment timing by using a timer and correct inhaler technique. Missed sessions will be rescheduled according to the participant’s availability to improve patient adherence. The intervention will combine 2 BD compounds: ipratropium (20 µg) and fenoterol (50 µg). Ipratropium is a short-acting antimuscarinic agonist, and fenoterol is a short-acting β-2 agonist BD. The BD or placebo will be delivered to the patient via a pressurized metered-dose inhaler with a spacer at least 20 minutes prior to CWRET, and CWRET will be performed no more than 1 hour after the administration of BD or placebo.

### BD and Placebo Delivery

The participants will be asked for total exhalation, followed by appropriate placement of the spacer mouthpiece with a nasal clip, and the first 8 puffs (30-second intervals between puffs, controlled by a timer) will be delivered through the opposite end of the spacer. The participants will be instructed to perform 5 tidal volume breaths for each puff. The total dose will consist of 160 µg of ipratropium and 400 µg of fenoterol.

### Outcomes

#### Primary Outcome

The primary outcome will be T_lim_ in CWRET. T_lim_ has been proposed as the main variable for assessing responsiveness to BDs in patients with chronic respiratory diseases [33]. A minimum clinically important difference >60 seconds has been suggested from improvements in clinical outcomes in BD trials [33,35]. T_lim_ will be determined by the inability to maintain a pedaling frequency below 60 rpm for more than 10 seconds despite verbal encouragement.

#### Secondary Outcomes

The following variables will be obtained from optoelectronic plethysmography: total chest wall and compartmental (upper ribcage, lower ribcage, and abdominal) volumes and TAA values. Thoracoabdominal kinematics will be compared during BD and the placebo effect. The associations between thoracoabdominal kinematics and CWRET variables will be determined.

#### Additional Measures

To appropriately describe the individuals with NCFB at baseline, basic demographic data and medical information, including age, smoking status, ethnicity, sex, anthropometric (height and weight) measures, and BD use, will be recorded. All reported clinical data will be confirmed in the hospital medical record review.

### Data Analysis

A sample of 39 participants was estimated as the number needed to provide 80% power to detect a between-group difference of 55.2 (SD 119.4) seconds in T_lim_, according to a pilot study (n=10; 80% females; 47.90 [SD 10.95]; FEV1 57.25% [SD 35.08%] predicted; FVC 69.12% [SD 31.21%] predicted; residual volume/total lung capacity 196% [SD 18.01%] predicted). The final sample size will be set at 45 patients, assuming up to a 10% loss during follow-up. The results will be analyzed according to the intention-to-treat principle to preserve the effects of group allocation and to provide an assessment of the practical impact of the intervention [38], as recommended by the CONSORT (Consolidated Standards of Reporting Trials) statement [39] (Multimedia Appendix 1). A median substitution will be imputed with the missing data. Data distribution will be assessed via the Kolmogorov‒Smirnov test. Comparisons between BDs and placebo data will be analyzed via 1-sided *t* tests or Wilcoxon tests for T_lim_ and repeated-measures analysis of variance or Friedman tests for thoracoabdominal kinematics variables. The number of participants who achieved the minimum clinically important difference (>60 seconds) in CWRET between BDs and placebo will be compared via chi-square tests. Pearson or Spearman correlation will be used to verify the associations between CWRET and thoracoabdominal kinematics variables. *P* values <.05 indicate statistical significance. The carryover, period, and sequence effects for T_lim_ will be estimated using generalized estimating equations models. If the carryover effect is not significant, a reduced model excluding the carryover term will be refitted. The statistical analysis will be performed using SPSS software (version 17.0; IBM Corp).

## Results

This study was funded by São Paulo Research Foundation (FAPESP, grants 2018/17788-3) and Conselho Nacional de Desenvolvimento Científico e Tecnológico (grant 312.279/2018-3) for the CardioO2 system (Medical Graphics Corporation) and the optoelectronic plethysmography system, respectively. The ethics approval (3.008.886) was granted in November 2018. The pilot study commenced in April 2019 but was interrupted due to the COVID-19 pandemic in early 2020; it was restarted and completed in April 2022. As of December 2023, enrolling by invitation is the last status of Clinical Trials.gov PRS (NCT05183841). Consent was obtained from 39 patients until May 2025. Of the 39 consented patients, one was excluded due to difficulty performing the cycle ergometer test and 6 patients did not complete the protocol. Data collection is expected to continue until November 2025. Data analysis is anticipated to begin in 2025, with initial results anticipated to be reported on and published after the completion of data collection. The publication of results is anticipated to be in 2025-2026.

## Discussion

### Principal Findings and Comparison to Prior Work

This randomized, controlled, double-blind crossover trial is intended to test the effect of dual bronchodilation on the exercise capacity of individuals with NCFB. In clinical practice, BDs have been recommended to treat only symptomatic individuals with NCFB [4]. Despite the common use of BDs in this population, there are no reports of their effects on the patient’s exercise capacity. A previous study showed no difference in the 6-minute walking test results of individuals with NCFB after a 6-month BD treatment (tiotropium) compared to the control group [13]. However, the exercise capacity was not the primary end point of this trial, and the 6-minute walking test is not the most appropriate choice for evaluating that outcome.

BDs have long been recommended to reduce exercise-induced bronchoconstriction and exercise limitation in patients with asthma and COPD, respectively [7,8]. According to the Global Initiative for Chronic Obstructive Lung Disease [8], BDs are recommended for individuals with severe disease, symptoms, or exercise limitations, as BDs reduce airway resistance, promote lung deflation, and enable better alveolar ventilation at rest and during exercise. Furthermore, dual bronchodilation is associated with better physical endurance capacity, exercise capacity, and physical activity level in individuals with COPD [40]. Additionally, TAA is very prevalent in individuals with COPD and is associated with exercise limitations [15,16]. The main effect of BDs on the ventilatory capacity is an increase in basal inspiratory capacity and a reduction in dynamic hyperinflation during exercise, enhancing exercise performance [17]. Most individuals with NCFB have airflow obstruction [41], air trapping, and diffusion impairment [42,43]; however, no studies have evaluated TAA or thoracoabdominal kinematics of patients with NCFB. Extrapulmonary features such as peripheral muscle endurance, exercise capacity, and health status are also affected by NCFB [44]. Hence, it seems plausible that BDs may improve the exercise capacity of individuals with NCFB, similar to what has been observed in individuals with COPD. Therefore, we hypothesize that dual bronchodilation can improve the exercise capacity of individuals with NCFB and reduce TAA during exercise.

### Strengths and Limitations

The strengths of this study include patient randomization and the crossover design, which reduce variability and increase statistical power. Besides, this is the first study to assess the exercise capacity and thoracoabdominal kinematics simultaneously of individuals with NCFB. Taken together, the results may highlight the effect of BD on exercise capacity and clarify a possible mechanism associated with it. The main limitation of this study is that patients will be recruited from a single referral hospital, and individuals with long-term oxygen therapy will be excluded. Unfortunately, we had to exclude patients with long-term oxygen therapy because our CPET equipment does not assess oxygen consumption with supplemental oxygen use, as it requires a special component. These limitations reduce the external validity of our results.

### Future Directions and Conclusion

Clinicians and researchers agree that the effect of BD in patients with NCFB and information in this field is urgently needed. If successful, this study will demonstrate that BDs can improve the exercise capacity in adults with NCFB and reduce TAA during exercise. Since exercise capacity reflects a patient’s functional status, future studies may explore whether its optimization can enhance independence and the quality of life in this population.

## Appendix

Multimedia Appendix 1 CONSORT checklist.

## Abbreviations

BD: bronchodilator
CONSORT: Consolidated Standards of Reporting Trials
COPD: chronic obstructive pulmonary disease
CPET: cardiopulmonary exercise test
CWRET: constant work-rate exercise test
FACED: FEV1% predicted score, Age, Chronic colonization by Pseudomonas aeruginosa, Radiological extension, Dyspnea
FEV1: forced expiratory volume in the first second
FVC: forced vital capacity
NCFB: non-cystic fibrosis bronchiectasis
SpO2: peripheral oxygen saturation
TAA: thoracoabdominal asynchrony
Tlim: time to the limit of tolerance
Wpeak: peak work rate
