# Supplementary material for: Effect of Dual Bronchodilation on the Exercise Capacity of Individuals With Non-Cystic Fibrosis Bronchiectasis: Protocol for a Randomized Controlled Double-Blind Crossover Study
Source: JMIR Res Protoc. 2025 Jul 28;14:e68582. doi: 10.2196/68582 (PMC12340461; doi:10.2196/68582)
Supplement: Multimedia Appendix 1 [file resprot_v14i1e68582_app1.pdf]

Table 1 CONSORT checklist of information to include when reporting randomised crossover trials

Section/topic	Item No	Description	Reported on Page Number/Line Number	Reported on Section/Paragraph
Title†	1a	Identification as a randomised crossover trial in the title	p. 1	Title
Abstract†	1b	Specify a crossover design and report all information outlined in *table 2*	p. 2 line 28	Abstract
**Introduction**				
Background‡	2a	Scientific background and explanation of rationale	p.3-5; lines 59-107	Introduction
Objectives‡	2b	Specific objectives or hypotheses	p.5; lines 105-107	Objective
**Methods**				
Trial design†	3a	Rationale for a crossover design. Description of the design features including allocation ratio, especially the number and duration of periods, duration of washout period, and consideration of carry over effect	p.5; line 112-113<br>p.8; line 181-184	Study desig;<br>Experimental design
Change from protocol‡	3b	Important changes to methods after trial commencement (such as eligibility criteria), with reasons	Not applicable for a protocol	Not applicable for a protocol
Participants‡	4a	Eligibility criteria for participants	p.7; line 156-167	Eligibility criteria
Settings and location‡	4b	Settings and locations where the data were collected	p.7; line 152	Study setting
Interventions†	5	The interventions with sufficient details to allow replication, including how and when they were actually administered	p.12; line 268-293	Interventions
Outcomes‡	6a	Completely defined prespecified primary and secondary outcome measures, including how and when they were assessed	p.13; lines 295-309	Outcomes
Changes to outcomes‡	6b	Any changes to trial outcomes after the trial commenced, with reasons	Not applicable for a protocol	Not applicable for a protocol
Sample size†	7a	How sample size was determined, accounting for within participant variability	p.14; line 318-322	Data analysis
Interim analyses and stopping guidelines‡	7b	When applicable, explanation of any interim analyses and stopping guidelines	No interim analysis were performed	No interim analysis were performed

Randomisation:				
Sequence generation‡	8a	Method used to generate the random allocation sequence	p.11; lines 260-263	Allocation, randomization, and blinding
Sequence generation‡	8b	Type of randomisation; details of any restriction (such as blocking and block size)	p.11-12; lines 258-266	Allocation, randomization, and blinding
Allocation concealment mechanism‡	9	Mechanism used to implement the random allocation sequence§ (such as sequentially numbered containers), describing any steps taken to conceal the sequence until interventions were assigned	p.11-12; lines 258-266	Allocation, randomization, and blinding
Implementation†	10	Who generated the random allocation sequence,§ who enrolled participants, and who assigned participants to the sequence of interventions	p.11; line 261	Allocation, randomization, and blinding
Blinding‡	11a	If done, who was blinded after assignment to interventions (for example, participants, care providers, those assessing outcomes) and how	p.12 lines 265-266	Allocation, randomization, and blinding
Similarity of interventions‡	11b	If relevant, description of the similarity of interventions	p.12; lines 264-265	Allocation, randomization, and blinding
Statistical methods†	12a	Statistical methods used to compare groups for primary and secondary outcomes which are appropriate for crossover design (that is, based on within participant comparison)	p.14-15; lines 317-336	Data analysis
Additional analyses‡	12b	Methods for additional analyses, such as subgroup analyses and adjusted analyses	p.14; lines 328-330	Data analysis
**Results**				
Participant flow (a diagram is strongly recommended)†	13a	The numbers of participants who were randomly assigned, received intended treatment, and were analysed for the primary outcome, separately for each sequence and period		
Losses and exclusions†	13b	No of participants excluded at each stage, with reasons, separately for each sequence and period		
Recruitment‡	14a	Dates defining the periods of recruitment and follow-up		
Trial end‡	14b	Why the trial ended or was stopped		
Baseline data†	15	A table showing baseline demographic and clinical characteristics by sequence and period		

Numbers analysed†	16	Number of participants (denominator) included in each analysis and whether the analysis was by original assigned groups		
Outcomes and estimation†	17a	For each primary and secondary outcome, results including estimated effect size and its precision (such as 95% confidence interval) should be based on within participant comparisons.¶ In addition, results for each intervention in each period are recommended		
Binary outcomes‡	17b	For binary outcomes, presentation of both absolute and relative effect sizes is recommended		
Ancillary analyses‡	18	Results of any other analyses performed, including subgroup analyses and adjusted analyses, distinguishing prespecified from exploratory		
Harms†	19	Describe all important harms or untended effects in a way that accounts for the design (for specific guidance, see CONSORT for harms32)		
**Discussion**				
Limitations†	20	Trial limitations, addressing sources of potential bias, imprecision, and if relevant, multiplicity of analyses. Consider potential carry over effects		
Generalisability‡	21	Generalisability (external validity, applicability) of the trial findings		
Interpretation‡	22	Interpretation consistent with results, balancing benefits and harms, and considering other relevant evidence		
**Other information**				
Registration‡	23	Registration number and name of trial registry	p.3	Trial registration
Protocol‡	24	Where the full trial protocol can be accessed, if available		
Funding‡	25	Sources of funding and other support (such as supply of drugs), role of funders	p.17	Acknowledgments

† Modified original CONSORT item.

‡ Unmodified CONSORT item.

§ Random sequence here refers to a list of random orders, typically generated through a computer program. This should not be confused with the sequence of interventions in a randomised crossover trial, for example receiving intervention A before B for an individual trial participant.

¶ A within participant comparison takes into account the correlation between measurements for each participant because they act as their own control, therefore measurements are not independent.
